# Changes of functional connectivity in the left frontoparietal network following aphasic stroke

**DOI:** 10.3389/fnbeh.2014.00167

**Published:** 2014-05-12

**Authors:** Dan Zhu, Jingling Chang, Sonya Freeman, Zhongjian Tan, Juan Xiao, Ying Gao, Jian Kong

**Affiliations:** ^1^Department of Neurology, Dongzhimen Hospital, Beijing University of Chinese MedicineBeijing, China; ^2^Department of Psychiatry, Massachusetts General Hospital, Harvard Medical SchoolBoston, MA, USA; ^3^Department of Radiology, Dongzhimen Hospital, Beijing University of Chinese MedicineBeijing, China

**Keywords:** aphasia, functional connectivity, independent component analysis, network connectivity analysis, the left frontoparietal network

## Abstract

Language is an essential higher cognitive function supported by large-scale brain networks. In this study, we investigated functional connectivity changes in the left frontoparietal network (LFPN), a language-cognition related brain network in aphasic patients. We enrolled 13 aphasic patients who had undergone a stroke in the left hemisphere and age-, gender-, educational level-matched controls and analyzed the data by integrating independent component analysis (ICA) with a network connectivity analysis method. Resting state functional magnetic resonance imaging (fMRI) and clinical evaluation of language function were assessed at two stages: 1 and 2 months after stroke onset. We found reduced functional connectivity between the LFPN and the right middle frontal cortex, medial frontal cortex, and right inferior frontal cortex in aphasic patients as compared to controls. Correlation analysis showed that stronger functional connectivity between the LFPN and the right middle frontal cortex and medial frontal cortex coincided with more preserved language comprehension ability after stroke. Network connectivity analysis showed reduced LFPN connectivity as indicated by the mean network connectivity index of key regions in the LFPN of aphasic patients. The decreased LFPN connectivity in stroke patients was significantly associated with the impairment of language function in their comprehension ability. We also found significant association between recovery of comprehension ability and the mean changes in intrinsic LFPN connectivity. Our findings suggest that brain lesions may influence language comprehension by altering functional connectivity between regions and that the patterns of abnormal functional connectivity may contribute to the recovery of language deficits.

## Introduction

Stroke-related aphasia is a significant clinical problem persisting in one third of acute stroke patients and one fifth of chronic stroke patients (Wade et al., 1986; Berthier, 2005). Identifying the brain mechanisms underlying stroke-related aphasia is critical for understanding its prognosis and developing new therapeutic methods to treat it. In addition to local dysfunction, stroke injury to certain locations of the brain can produce specific as well as local and network dysfunction. In order to understand the influence of an individual cortical lesion, we must consider not only the loss of local neural function, but also the lesion-induced changes in the larger network interactions in the brain.

Functional segregation and integration are two major organizational principles of the human brain. An optimal brain requires a balance between local specialization and global integration of brain functional activity. However, recent work has mainly focused on defining the contribution of individual elements (e.g., inferior frontal gyrus, anterior temporal lobe) in the network. Understanding connectivity within a whole network is critical both to understanding its normal function and to explaining brain recovery (Catani et al., 2005). The function of any brain region cannot be understood in isolation but only in conjunction with the regions with which it interacts (Seghier et al., 2010).

Language is an essential higher cognitive function supported by large-scale brain networks. The superior temporal cortex (Wernicke's area) and the inferior frontal cortex (Broca's area) have been classically associated with language comprehension and production. Saur et al. (2008) identified two routes connecting the frontal and temporal language regions; a dorsal route associated with phonological processing and a ventral route associated with semantic processing. Additionally, lesion and fMRI studies (Dronkers et al., 2004; Price, 2010) have identified additional temporal, parietal, and prefrontal regions, supporting the involvement of a more extended language network (Mesulam, 1990; Turken and Dronkers, 2011). Damage to these networks (e.g., the frontal-temporal network and the frontal-parietal network) often leads to the impairment of language function, but patients frequently recover some or all of their abilities. The recovery time of aphasia varies from months to years, suggesting that recovery of language function following stroke is unpredictable (Pedersen et al., 1995).

In this study, we investigated the LFPN in aphasic patients by integrating an ICA approach and a network connectivity analysis method to explore how network embedding influences a region's functional role and the consequences of its being damaged. We focus on this network due to its strong association with language-cognition paradigms that are consistent with Broca's and Wernicke's areas (Smith et al., 2009). We aimed to assess the significance of functional connectivity by measuring the relationship between functional connectivity of the LFPN and performance deficits in stroke patients. More specifically, we investigated whether the degree of disruption in LFPN functional connectivity correlated with the severity of behavioral deficits at the acute stage and whether this correlation was maintained over the course of recovery.

We also aim to investigate the role of functional connectivity changes of LFPN in the recovery process following stroke. In a recent study, Park et al. (2011) reported dynamic changes in the lateralization of functional connectivity of motor networks in the first 6 months post stroke, where measures of functional connectivity at stroke onset were found to be positively correlated with motor outcomes. Studies from other groups also suggest that functional connectivity can be associated with treatment-induced behavioral changes in aphasia (Price et al., 2006; Marcotte et al., 2013). Overall, investigating the dynamic changes of functional connectivity and its association with the clinical outcomes will enhance our understanding of the relationship between human brain function and behavior (Corbetta et al., 2005; Thiebaut de Schotten et al., 2005; Sharp et al., 2010b).

## Methods

### Subjects

We recruited 14 right-handed patients [13 males, age ranging from 34 to 67 years with mean (*SD*): 49.4 (10.7) years] with a diagnosis of aphasia following left hemisphere stroke from the Department of Neurology at Dongzhimen Hospital over the course of 14 months (March 2012–April 2013). Experienced neurologists performed clinical assessments to confirm the diagnoses of aphasia. These assessments were based on a comprehensive evaluation, including neurological history and examination, language assessment, and structural routine MRI. The local Medical Ethical Review Board approved the protocol and we obtained written consent from all subjects prior to all experimental proceedings.

The patients we enrolled were native Chinese speakers who were right handed, as determined by the Edinburgh Handedness Inventory (EHI) score ≥50 (Oldfield, 1971). All patients had single unilateral left-hemisphere stroke and a diagnosis of aphasia based on a standardized language test from the Chinese Rehabilitation Research Center Standard Aphasia Examination (CRRCAE) (Zhang et al., 2005). All patients received a score of ≥2 on the Boston Diagnostic Aphasia Examination (BDAE) severity rating scale, which indicated that they could converse about familiar topics with help from the listener, but had trouble conveying their ideas (Love and Oster, 2002) and a score of <3 on the modified Rankin scale, which indicated that they were moderately disabled, but able to walk without assistance (van Swieten et al., 1988). Patients were excluded from the study if they had a history of other neurological or psychiatric disorders and/or an inability to enter the MRI scanner because of non-MRI compatible prostheses.

We recruited 14 participants matched in age, gender and educational level as controls from communities near the hospital [13 males, age ranging from 34 to 67 years with mean (*SD*): 49.4 (10.7) years]. All subjects in the control group reported no history of neurological or psychiatric illness and were not taking regular medication.

We invited all patients to participate in two fMRI scan sessions and all healthy controls to participate in one fMRI scan session. We collected the patients' first fMRI scan (time point one) 1 month after stroke onset and the second scan (time point two) 2 months after stroke onset. We administered the CRRCAE tests before each scan.

### Language assessment

Prior to each fMRI scan, we administered the CRRCAE to evaluate the degree of language impairment for each patient. This scale was developed for clinical evaluation and therapy, combining the syntactic and lexical characters in Chinese. The reliability and validity of this scale has been tested in a previous study (Zhang et al., 2005). The CRRCAE includes nine tests and 30 subtests producing a standardized score based on correct responses. The scale can test three aspects of language ability: comprehension, production, and other abilities related to language skills. In this study, we focused on the absence of comprehension and production abilities; thus, we included only comprehension (auditory and reading comprehension) and production scores (repetition, naming, and overt reading) in the correlation analysis. We used SPSS for correlation analysis. For those groups with small sample sizes (*n* < 10), we applied Spearman correlation, a nonparametric method.

### Data acquisition

We performed all brain imaging on a 3T Siemens TRIO system. We used a 12-channel head coil with foam padding to restrict head motion. For resting state fMRI, we used a gradient-echo echo-planar sequence sensitive to blood oxygenation level-dependent (bold) contrast (TR/TE = 2000/30 ms, FOV = 225 × 225 mm^2^, flip angle = 90°, voxel size = 3.5 × 3.5 × 3.5 mm^3^). We collected 31 slices with 3.5 mm thickness and a 0.7 mm gap. Each fMRI scan lasted 6 min and 6 s. The first 8 s were dummy scans, discarded from data analysis. Thus, we collected 179 image volumes in total. In addition, we used a high-resolution T1-weighted scan [repetition time (TR)/echo time (TE) = 1900/2.13 ms, field-of-view (FOV) = 256 × 256 mm^2^, flip angle = 9°, acquired voxel size = 1.0 × 1.0 × 1.0 mm^3^] for anatomical localization.

### Patient lesion mapping

We constructed a lesion overlap image for all 14 patients. A manually drawn outline of the lesion on the T1 image of each patient was used to overlap on the average structure image using Turtleseg (http://www.turtleseg.org/).

### ICA analysis of resting state data

We performed data analyses using MELODIC of FMRIB Software Library (FSL version 5.0.1; www.fmrib.ox.ac.uk/fsl) to identify large-scale patterns of temporal signal-intensity coherence, interpreted as functional connectivity, in the population of subjects (Beckmann et al., 2005). Preprocessing of functional images consisted of the removal of non-brain tissue, motion correction, temporal band-pass filtering at 0.01 to 0.1 Hz, spatial smoothing using a 8 mm full-width at half-maximum Gaussian kernel, and 8-parameter nuisance signal extraction. To coregister fMRI images to a standard space, we first registered functional images to each individual's high-resolution T1 anatomical scan, and further registered them to the MNI152 template using linear affine transformations with 12 degrees of freedom.

We performed probabilistic independent component analysis (PICA) at low dimensionality (20 components) to derive the group's (*n* = 26) resting state networks. We based the network identification on their spatial similarity to functional networks described in earlier studies (Damoiseaux et al., 2006; Smith et al., 2009; Biswal et al., 2010) and calculated the cross correlation between our group-level networks and the LFPN template networks derived from 1414 healthy subjects (Biswal et al., 2010). We assigned the group-derived networks that showed the highest spatial overlap with the template network to that particular functional network.

In this study, we identified the LFPN as the a priori network for further analysis. We chose this network because it is associated with language-cognition function (Smith et al., 2009). We carried out a voxel-wise comparison of the resting functional connectivity using a regression technique, referred to as the “dual-regression” approach (Filippini et al., 2009).

We used spatial maps of the group ICA in a linear model fit against each individual fMRI data set (spatial regression) to create matrices that described the temporal dynamics for each component and subject separately. We used these matrices in a linear model fit against the associated subject's fMRI data set (temporal regression) to estimate subject-specific spatial correlation maps. After this dual regression, we collected spatial maps of all subjects into single 4-dimensional files for each original independent component. We used nonparametric permutation tests to detect statistically significant differences between the groups within the boundaries of the spatial maps obtained with MELODIC. We performed all analyses with a voxel-wise cluster forming threshold of *Z* > 2.3 and a corrected cluster significance threshold of *P* < 0.05. We used the regions that showed significant differences between groups [spherical regions of interest (ROIs) were centered on the MNI coordinate of the cluster peaks, and with a radius of 4 mm] to extract mean *z*-values from each individual spatial map, including time points one and two (FWE-corrected *P* < 0.05) for the correlation analysis using SPSS (version 16.0; SPSS, Chicago, IL, USA). Then we performed correlation analyses to assess association between the ROIs within the LFPN and language test results.

### Network connectivity analysis of left frontoparietal network

**Figure 1:**
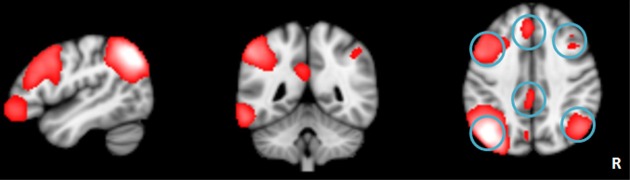
**The left frontoparietal network identified by independent component analysis**.

To better understand the impact of network connectivity differences on whole-brain intrinsic connectivity, we employed network connectivity analysis to LFPN changes. We used standard image processing methods with SPM8 (http://www.fil.ion.ucl.ac.uk/spm) and the conn toolbox (http://www.nitrc.org/projects/conn) for functional connectivity and network connectivity analysis. Our pre-processing steps included correcting for motion, coregistering with the anatomic scan, normalizing into the Montreal Neurological Institute space, resampling at 2 mm^3^, and smoothing with a Gaussian kernel of 6 mm^3^ full-width at half maximum. We extracted the bold time series data for six ROIs within the LFPN obtained from the ICA analysis mentioned above. The ROIs were derived from the LFPN (centered on the MNI coordinate of the cluster peaks, and with a radius of 4 mm) (Figure 1). Prior connectivity studies have employed similar approaches to the investigation of the default mode network (DMN) (Dosenbach et al., 2007; Fair et al., 2008; Posner et al., 2013). The LFPN ROIs and their coordinates are delineated in Table 1. We correlated the time series data for each ROI region by region for each subject, producing a single 6 × 6 correlation matrix for each subject. We calculated the mean index of the LFPN by reducing each subject's 6 × 6 correlation matrix from the overall mean into a single variable that indexed the global connectivity for the LFPN including all edges. We compared the mean network connectivity index across the patient and control groups using a two-sample *t*-test. We also performed correlation analyses to assess the relationship between the mean connectivity of the LFPN and the language test results.

**Table 1:** **The left frontoparietal network regions**.

**Regions**	**MNI Coordinates**		
	***x***	***y***	***z***
Left precuneus	−40	−70	46
Left middle frontal gyrus	−32	20	46
Right superior parietal gyrus	42	−66	50
Right middle frontal gyrus	38	20	44
Posterior cingulate gyrus	−2	−34	30
Left medial frontal gyrus	−4	38	34

## Results

### Demographics and language performance

**Figure 2:**
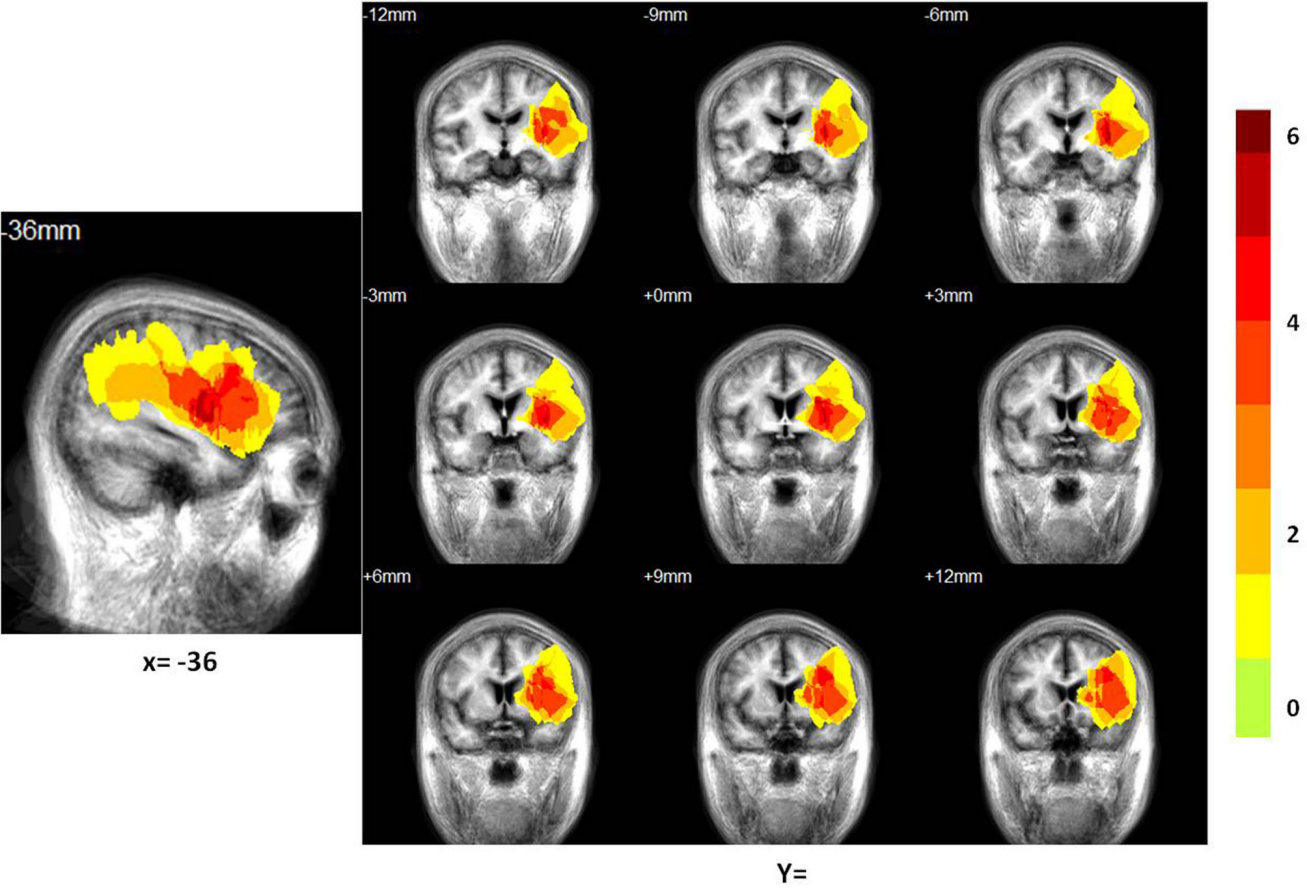
**Distribution of the lesion areas of all patients with aphasia, on the average patients' structure brain template**. The intensity scale refers to the maximum number of patients with lesions at a particular voxel.

The locations of the patients' infarcts are shown in Figure 2. Of the total 28 subjects (14 patients) enrolled in the study, we dropped one control subject due to technical issues in brain structure and one patient due to failure in the pre-processing of the ICA and network connectivity analysis. We included 13 patients and 13 controls in the ICA and network connectivity analyses. Of all 13 patients, eight patients completed the second scan. Six patients could not participate in the second scan due to their inability to return to the hospital. For characteristics of these patients and scores from all subtests of the CRRCAE, see Table 2.

**Table 2:** **Patient characteristics and subtest results of the CRRCAE at time point one and two**.

**Patient number**	**Gender**	**Age**	**Handedness**	**Aphasia profile**	**Comprehension score**	**Production score**	**Type of stroke**	**Site of lesion**	**BDAE severity rating scale**	**Modified Rankin scale**
1	M	41	100	Broca's	60/80	80/90	Ischemia	Frontal, parietal	4	0
2	M	35	100	Broca's	70/75	53/83	Hemorrhage	Striatocapsular	3	2
3	M	55	100	Anomic	40/71	51/81	Ischemia	Striatocapsular	2	2
4	M	34	100	Broca's	65/75	35/66	Hemorrhage	Striatocapsular	3	2
5	M	59	100	Global	10/54	43/72	Ischemia	Frontal, temporal, insular	2	1
6	M	51	100	Global	32/72	55/84	Ischemia	Frontal, parietal	2	2
7	M	58	90	Global	20/NA	60/NA	Ischemia	Parietal, occipital	2	1
8	M	36	100	Global	33/NA	75/NA	Ischemia	Frontal, parietal, temporal	3	1
9	M	44	90	Broca's	64/NA	82/NA	Ischemia	Striatocapsular	3	1
10	M	63	100	Broca's	68/NA	90/NA	Ischemia	Frontal, temporal, parietal	4	0
11	M	56	100	Broca's	75/80	85/90	Ischemia	Striatocapsular	4	0
12	M	48	100	Broca's	80/NA	90/NA	Ischemia	Striatocapsular	4	0
13	M	44	100	Broca's	70/80	65/85	Hemorrhage	Striatocapsular	2	2
14	F	67	100	Global	27/NA	50/NA	Ischemia	Frontal, temporal	2	2

*M, male; F, female; Handedness: 0 = left-handed in 10 items, 100 = right-handed in 10 items; Site of lesion is based on clinical report; Comprehension and production score are based on CRRCAE test at two time points separated by “/”, NA indicate the data is not available; BDAE severity rating scale: 0 = no usable speech or auditory comprehension, 5 = minimal discemible speech handicaps, patient may have subjective difficulties that are not apparent to listener; Modified Rankin scale: 0 = no symptoms at all, 6 = dead*.

The comprehension subtest scores that we calculated consist of the combined auditory and reading comprehension scores of each subject and the production subtest score consists of the combined repetition, naming, and overt reading scores of each subject. At time point one (*n* = 14), the scores on the comprehension and production subtests were 51 (23) [mean (*SD*)] and 65 (18), respectively. At time point two (*n* = 8), the scores for comprehension and production were 73 (9) and 81 (8), respectively. The nonparametric rank test showed that at time point two, there was a significant increase in the comprehension (*z* = −2.527, *P* = 0.012) and production (*z* = −2.527, *P* = 0.012) score as compared to that at time point one. The average improvement of the CRRCAE scores of the patients on the comprehension and production subtests between the two time points were 21 (16) and 23 (10), respectively.

All patients received conventional stroke treatment, which involved a 30-min language therapy session that involved listening comprehension, reading comprehension, and verbal production training at least three times per week throughout the entire period of observation.

### Results from ICA analysis

**Figure 3:**
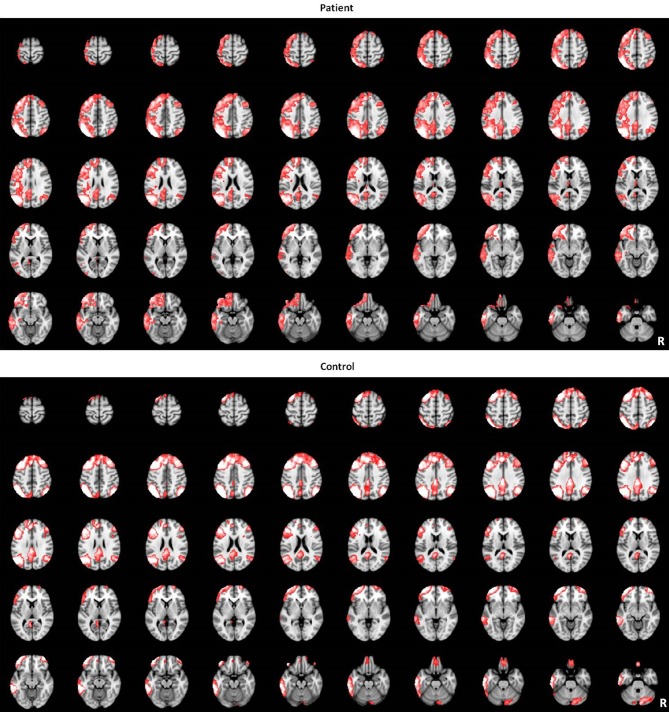
**The LFPN identified by group-level ICA in patients and controls groups**.

The LFPN that we obtained from our cohort of subjects includes the bilateral parietal cortex, bilateral frontal cortex, medial frontal cortex and posterior cingulate cortex (Figure 1). We obtained 20 spatial and temporal components from the ICA analysis and noted that the results from previous ICA analyses support the selection of the LFPN (Smith et al., 2009; Biswal et al., 2010). The group-level ICA was performed separately in patients and controls (Figure 3).

**Figure 4:**
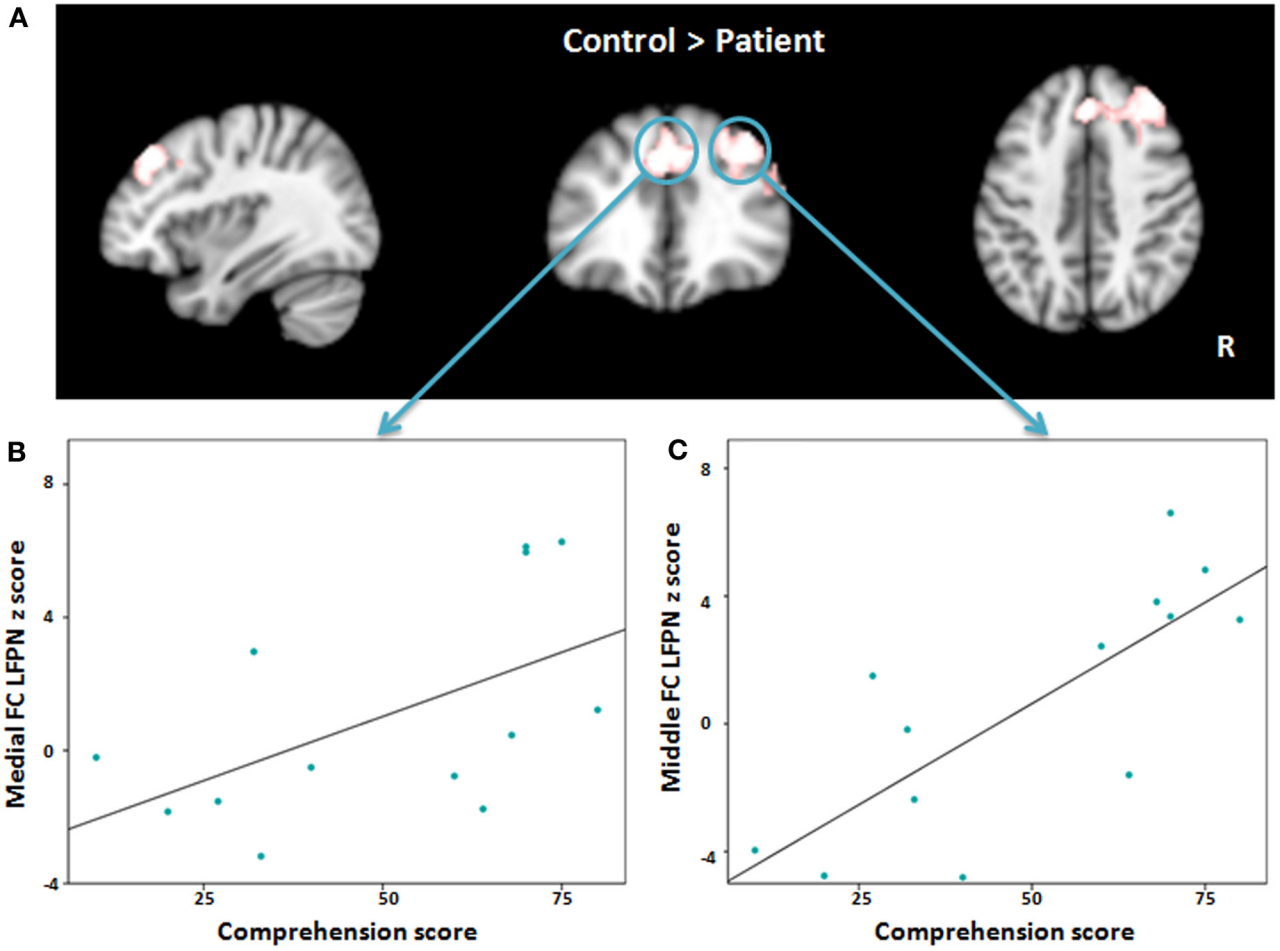
**(A)** Red-white heat map represents results of decreased functional connectivity within left frontoparietal network in patients compared with controls. **(B,C)** Scatter plots depict the relationship between abnormal regions functional connectivity and severity of comprehension deficit at time point one in patient group. FC, Frontal cortex; LFPN, left frontoparietal network.

When we compared the patients to the matched healthy controls, we found significantly reduced functional connectivity between the LFPN and the right middle frontal cortex, the medial frontal cortex, and the right inferior frontal cortex (Figure 4A, Table 3) in aphasic patients. No regions showed significantly increased functional connectivity in patients as compared to controls.

**Table 3:** **Decreased FC clusters in aphasic patients compared with controls (*P* < 0.05, using FWE correction at cluster level)**.

**Contrast**	**Brain region**	**Cluster size**	**MNI coordinates**			***z*-value**
			***x***	***y***	***z***	
Control > patient	Right middle frontal cortex	199	36	34	42	3.82
	Medial frontal cortex	149	0	30	42	3.8
	Right inferior frontal cortex	5	58	30	4	3.45

To explore the association between language impairment and lower regional functional connectivity within the LFPN, we applied a Pearson correlation between the regions that showed significant differences in the language production and comprehension scores separately at time point one. The results showed a significant association between the medial frontal cortex and comprehension score (*r* = 0.555, *P* = 0.049; Figure 4B) and strong correlation between the right middle frontal cortex and comprehension score (*r* = 0.781, *P* = 0.002; Figure 4C), such that lower intrinsic functional connectivity between the LFPN and right middle frontal cortex and medial frontal cortex coincided with greater impairment of comprehension ability in aphasic patients. To test whether this correlation was maintained over the course of recovery, we also measured the association between LFPN functional connectivity and the above two regions and the comprehension score at time point two using a Spearman correlation for eight patients. Our results showed a marginally significant association between the LFPN and the right middle frontal cortex connectivity and comprehension score (*r* = 0.728, *P* = 0.064). We found no significant association between these three regions and production score.

### Results from network connectivity analysis

**Figure 5:**
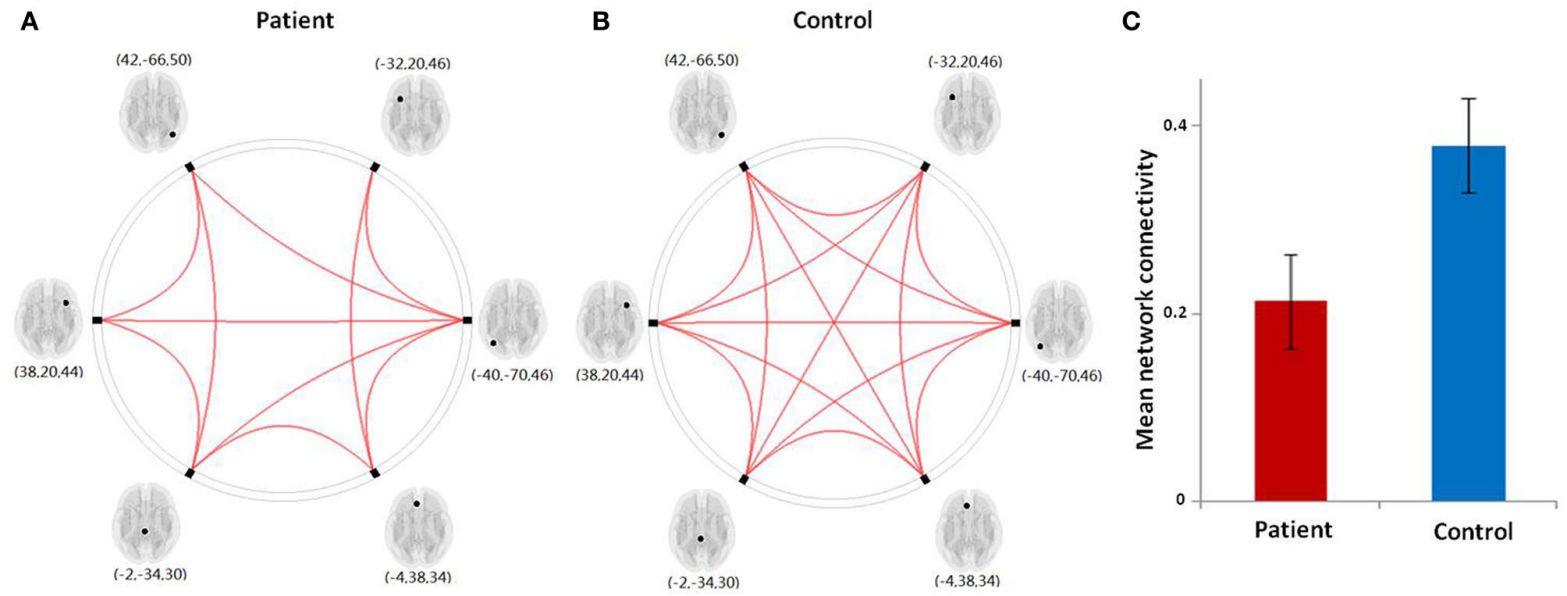
**(A,B)** Graphical presentation of the left frontoparietal network in aphasic patient and healthy control groups separately identified by network connectivity analysis analysis. A threshold of FDR-corrected *P* < 0.05 was applied. **(C)** The mean network connectivity index in two groups. The error bars indicate standard errors.

**Figure 6:**
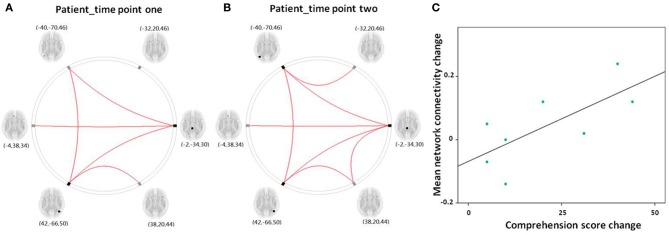
**(A,B)** Graphical presentation of the left frontoparietal network in aphasic patient in time point one and two separately identified by network connectivity analysis analysis. A threshold of FDR-corrected *P* < 0.05 was applied. **(C)** Scatter plots depict the relationship between the mean left frontoparietal network connectivity change and the comprehension score change in patient group between time point one and two.

The results from our network connectivity analysis suggest that patients and controls are associated with two different sets of network connectivity. At time point one, the network of the controls was considerably more intact than that of the patients (Figures 5A,B). The network showed a different pattern at time point one as compared to time point two (Figures 6A,B). At time point one, we observed significantly weaker LFPN connectivity in patients as compared to controls (mean network connectivity index of patients: 0.213 and controls: 0.379; *t* = 3.104, *P* = 0.005) (Figure 5C).

The Pearson correlation analysis showed a significant association between mean LFPN connectivity and comprehension score at time point one (*r* = 0.781, *P* = 0.002) in aphasic patients, indicating that decreased LFPN connectivity coincided with more severe the loss of comprehension ability.

For the eight patients that completed the two fMRI scans, the Spearman correlation analysis showed that the change in mean network connectivity index was marginally associated with the improvement of comprehension ability (*r* = 0.655, *P* = 0.078; Figure 6C). That is, patients who exhibited the highest level of comprehension improvement also showed the highest increase in mean LFPN network connectivity.

## Discussion

In this study, we integrated ICA and network connectivity analysis methods to investigate the association between resting state functional connectivity and language function in aphasic patients. We found reduced functional connectivity between the LFPN and the right middle frontal cortex and medial frontal cortex in aphasic patients. Stronger functional connectivity coincided with more preserved language comprehension ability after stroke. This strengthening in connectivity could be maintained over the course of recovery in the right middle frontal cortex. In addition, we found reduced LFPN connectivity in aphasic patients, as indicated by the mean network connectivity index of key regions in the LFPN. We associated the decreased LFPN connectivity with the impairment of language function in the comprehension ability of stroke patients. We also found significant association between the recovery of comprehension ability and the mean improvement in intrinsic LFPN connectivity.

Speech comprehension ability reflects a complex cognitive process, including attention, working memory, comprehension monitoring, and strategic behavior. In aphasic patients, we found decreased functional connectivity between the LFPN and the right middle frontal cortex, the medial frontal cortex, and the right inferior frontal cortex. Previous studies found that the medial prefrontal cortex is activated during task switching and performance monitoring and/or adjustments (DiGirolamo et al., 2001; Rushworth et al., 2002; Ridderinkhof et al., 2004; Wager et al., 2004; Crone et al., 2006). More specifically, using resting state connectivity over more than 1000 subjects, investigators have found that both subdivisions in the medial frontal cortex and posterior cingulate cortex are strongly connected to the inferior parietal lobe, a key region in language processing (Lambon Ralph, 2010). Historically, investigators have defined the dorsal, rather than the ventral, medial frontal cortex by its connection to the inferior parietal cortex (Luciana, 2001). This was not part of the LFPN we defined in this study. Our results showed that LFPN showed a significant association with dorsal medial prefrontal cortex, we speculate these regions may interact rather strongly during task-related processes (Tomasi and Volkow, 2012). Results from previous studies have also suggested that the medial prefrontal cortex is involved in coherence processing in language comprehension (i.e., establishing the pragmatic connection between successively presented sentences) (Ferstl and von Cramon, 2002). Our results support the idea that the medial prefrontal cortex plays an important role in language processing.

The left middle frontal cortex and the inferior frontal cortex are also critical components of language processing. Investigators have interpreted the involvement of the inferior frontal cortex in aphasic patients as reflecting the impairment of a working memory system for semantic information, whereas the middle frontal cortex has been attributed to deficits in the general cognitive control process (Turken and Dronkers, 2011). We believe that comprehension ability in patients could reflect the semantic cognition process. Results from a previous study suggest that lesions located in the left hemisphere after stroke can cause the right hemisphere to selectively contribute to the reorganization of language (Crinion and Price, 2005). This may be due to the disinhibition of the right hemisphere in the presence of left hemisphere lesions. Other studies have suggested that a relationship exists between lesion size and the success of hemispheric transfer, where larger lesions may result in a complete transfer of functions to the contralateral hemisphere. Conversely, in the presence of a smaller lesion, intact areas of the damaged hemisphere may inhibit complete transfer (Grafman, 2000). We speculate that in our study, the lesions of patients may have not been large enough, so that the right hemisphere was still inhibited by the intact remains in the left hemisphere. This may explain why we found decreased functional connectivity in the homologous parts of these two regions in the LFPN. We also found decreased connectivity in the middle frontal cortex to be associated with cognitive function and patients maintained this correlation over the course of a 1-month recovery. This finding is consistent with prior literature on the reorganization of language recovery after stroke.

Overall, we have found a multitude of evidence supporting our finding that the LFPN may be especially relevant to understanding cognitive impairment in aphasic patients. Considering the complexity of language processing, which includes semantic, lexical and phonological levels, motor programming, and access to visual and memory representations in oral naming, the network analysis approach seems particularly suitable for characterizing post-stroke recovery. Under the assumption that disconnection between the distal frontoparietal areas may underlie primary cognitive deficits, recent fMRI studies have explored the frontoparietal network and its association with performance in attention and working memory tasks (Sharp et al., 2010a,b). In our study, aphasic patients showed a significant decrease in mean LFPN connectivity as compared to controls and the magnitude of this decrease was correlated with language comprehension ability. This result may suggest that there was disrupted functional connectivity in the LFPN, which induced cognitive deficits at the acute stage of lesion to the left hemisphere.

After 1 month of recovery following stroke, mean LFPN connectivity seems to be associated with the recovery of language comprehension function. Increasing LFPN connectivity is likely to be a natural, intrinsic, and plastic neural mechanism for increased cognitive function and can be regarded as early language recovery. Specifically, investigators have found that increased frontoparietal integration during language task processing in patients who are in recovery following aphasia can be associated with the recovery of cognitive function (Sharp et al., 2010b). Reorganization of the functional network is essential to the recovery of language function. We found that increased LFPN connectivity corresponded with greater improvement in language function after 1 month of recovery following stroke. This result suggests that promoting this connectivity should be an important target for future research aimed at restoring language deficits.

Our study was not without limitations. The patients completing two fMRI scans received a conventional therapy, so we remain unsure about whether to attribute the changes in network functional connectivity to the medication or the natural recovery of language function. Additionally, many aphasic patients had multifocal brain lesions in the left hemisphere, which may have potentially complicated the interpretation of our results. Brain damage to the left hemisphere regions—the frontal or parietal cortex, this may cause functional network abnormalities that induce language function deficit. In all 13 patients, only two patients had wide lesions spreading to the frontal and parietal cortices and no more than three patients had a lesion in the same area of the left frontoparietal network. Still, it is reasonable to believe that the function of the LFPN was preserved in our sample of patients. Thus, studies with larger sample sizes that only enroll patients with unifocal lesions are necessary to verify our findings in the future.

In summary, we found functional connectivity abnormalities in the LFPN in aphasic patients. Our results suggest that brain lesions may influence language comprehension ability by causing impairment of both functionality in affected regions and functional connectivity with other regions. Identifying the patterns of abnormal functional connectivity may contribute to therapies that enhance the recovery of language deficits and cognitive function following stroke.

### Conflict of interest statement

The authors declare that the research was conducted in the absence of any commercial or financial relationships that could be construed as a potential conflict of interest.
